# Cervical cancer risk perceptions, sexual risk behaviors and sexually transmitted infections among Bivalent Human Papillomavirus vaccinated and non-vaccinated young women in Uganda - 5 year follow up study

**DOI:** 10.1186/s12905-017-0394-y

**Published:** 2017-06-02

**Authors:** Edward Kumakech, Sören Andersson, Henry Wabinga, Caroline Musubika, Samuel Kirimunda, Vanja Berggren

**Affiliations:** 1 School of Biomedical Sciences, College of Health Sciences, Makerere University, P.O. Box 7072, Kampala, Uganda; 2 School of Health and Medical Sciences, Örebro University, 701 82 Örebro, Sweden; 3 Department of Laboratory Medicine, Örebro University Hospital, 703 62 Örebro, Sweden; 4 Department of Pathology, Kampala Cancer Registry, Makerere University, P.O. Box 7072, Kampala, Uganda; 5 Department of Medical Microbiology, Immunology Laboratory, Makerere University, P.O. Box 7072, Kampala, Uganda; 6 Department of Medical Microbiology, Immunology laboratory, Makerere University, P.O. Box 7072, Kampala, Uganda; 7 Department of Health Sciences, Lund University, 221 00 Lund, Sweden

**Keywords:** Bivalent Human Papillomavirus (HPV) vaccination, cervical cancer risk perceptions, sexual risk behaviors, Sexually transmitted infections, Young women, Uganda

## Abstract

**Background:**

Previous studies were conflicting regarding the associations between HPV vaccination, cervical cancer risk perceptions, high-risk sexual behaviors and STIs. This study compared the HPV-vaccinated and non-vaccinated young women in Uganda regarding cervical cancer risk perceptions, high-risk sexual behaviors, syphilis and HIV infections 5 years after vaccine implementation.

**Methods:**

This was a population-based comparative cross-sectional survey conducted in Uganda. The 438 participants were sexually active young women aged 15–24 years and mean age was 18.6 (SD 1.4). The majority (53.0%) were HPV-vaccinated in 2008 without assessment of sexual activity prior to HPV vaccination. Upon verbal assessment of sexual activity at the time of follow-up, data were collected using a questionnaire and laboratory testing of blood samples for syphilis and HIV infections.

**Results:**

There were no significant differences between the HPV-vaccinated and non-vaccinated groups regarding the prevalence of high-risk sexual behaviors, syphilis and HIV infections. Cervical cancer risk perceptions and age at sexual debut were nonetheless significantly lower among the vaccinated group compared to their non-vaccinated counterparts. However, HPV vaccination was not significantly associated to cervical cancer risk perceptions and early age at sexual debut in multivariate logistic regression analysis.

**Conclusions:**

We found no associations between HPV vaccination, cervical cancer risk perceptions, high-risk sexual behaviors, syphilis and HIV infections among young women in Uganda 5 years after vaccine implementation. Young girls in the study population were found to be sexually active at a young age, affirming the importance of targeting girls of younger age for HPV vaccination.

**Electronic supplementary material:**

The online version of this article (doi:10.1186/s12905-017-0394-y) contains supplementary material, which is available to authorized users.

## Background

Cervical cancer (CC) is the third most common cancer among women globally in terms of 5-year prevalence (i.e. total cases identified over a 5-year period) with an estimated 527,624 new cases and 265,672 deaths in 2012 [1]. About 87% of the CC deaths occur in less developed countries. In East Africa, CC is the most common cancer and also the leading cause of cancer related death among women [1]. In Uganda, the age-standardized incidence rate for CC was estimated to stand at 44.4/100,000 women person years in 2012 which was one of the highest in the world [1].

An infection with sexually transmittable Human papillomavirus (HPV) is a necessary but not a sufficient cause of cervical cancer [2]. There are 14 so called high risk HPV (HR-HPV) genotypes associated with CC which include HPV 16/18 the antigens included in the currently available HPV vaccines [3]. Notably, 70% of cervical cancer cases are attributable to HPV16/18 alone, making the use of the two available HPV vaccines justifiable because both of them contain antigens for HPV16/18. In Uganda, HPV vaccination programme started in 2008 as a pilot project in two districts namely Ibanda and Nakasongola districts. The vaccine in use during the pilot phase was the bivalent HPV vaccine (Cervarix®, GlaxoSmithKline, Belgium) which administered to girls aged 10 years or in primary school grade 5 in 3 doses at an interval of 0, 1 and 6 months. The health workers take the vaccine to the targeted girls in schools as part of school health programme. In addition to school visits, community visits were also conducted to ensure eligible out of school girls also gets vaccinated. A phased approach to national rollout of HPV vaccination using the quadrivalent HPV vaccine (Gardasil, Merck MSD, USA) followed suit in 2010 initially in 12 districts and countrywide by 2014. Nationally, the vaccine is administered to girls aged 9 years or in primary school grade 4 in 2 doses at an interval of 0 and 6 months. The delivery of the HPV vaccines to the girls is integrated into a bi-annual (every April and October) school health programme popularly known as child health days plus (CHDP) during which health workers visits schools to provide catchup vaccination and medication against intestinal worms. For out of school girls, the delivery of the vaccine is integrated into monthly community outreaches for immunization services delivery. HPV vaccination coverage survey conducted in the study area (Ibanda) around the period of the study indicated HPV vaccination coverage of 95% [4]. At the time of the study, the Uganda ministry of health had no written strategic plan for use of 9-valent HPV vaccine instead of or concurrently with bivalent and quadrivalent vaccines.

Important to note is that HPV vaccine alone would not completely prevent cervical cancer because the vaccine contains only 2 of the 14 HR-HPV types associated to CC. Secondly, it would be impossible to attain 100% vaccination coverage particularly in developing countries with weak health systems. Thirdly, potential epidemiological changes may occur as a result of HPV vaccination. The changes are most likely to arise because there are other HR-HPV types that are not covered by the current vaccines in developing countries (bivalent and quadrivalent HPV vaccines) and so it is possible that epidemiological changes indeed may occur by transitioning to disease caused by HR-HPV types not covered by HPV vaccination . Such discussions of the limitations of the currently available HPV vaccines are frequent in the literature [5–7]. The aforementioned discussions on the limitations of the currently available HPV vaccines that target HPV16/18 for CC prevention underscores the importance of simultaneously addressing other determinants of CC in order to optimize the benefits of HPV vaccination. Cervical cancer risk perceptions, high-risk sexual behaviors and other sexually transmitted infections (STIs) such as syphilis and HIV are some of the critical determinants of HPV infections and CC that needs to be addressed alongside HPV vaccinations [8, 9].

The status of high-risk sexual behaviors, syphilis and HIV infections in Uganda at the time of the study shows a decreasing trend in the prevalence of the above determinants among the 15–24 year-old young women from the pre-HPV vaccine introduction period to post-HPV vaccine period. For example, the percentage of the 15–24 year-old young women who initiated sexual intercourse before the age of 15 decreased from 14.4% in 2005 to 10.9% in 2011 [10, 11]. Similarly, for syphilis, the percentage of the 15–24 year-old young women who were positive for syphilis decreased from 2.1% in 2005 to 1.4% in 2011 [10, 11]. The same decreasing trend in the prevalence was also reported for HIV infection whereby the percentage of the 15–24 year-old young women who were positive for HIV decreased from 4.3% in 2005 to 4.9% in 2011 [10, 11]. As for the trend of CC risk perceptions among young women in Uganda, previous studies specific to young women couldn’t be found. Cervical cancer risk perceptions are often measured using a composite score calculated from a scale comprising of many items and therefore, point estimates also are not readily available for young women in Uganda.

Regarding the association between HPV vaccination and high risk sexual behaviors, previous studies were conflicting in their findings. Specifically, there were previous studies including in US and Canada that indicated no association between HPV vaccination, risk perceptions and high-risk sexual behaviors among young women [12–18]. For example, a study conducted among 18–30 year old Australian young women reported that attitudes to safe sexual behavior were the only factors significantly associated to vaccination status such that vaccinated women held more positive attitudes to practicing safe sexual behaviors [14]. Similarly, a short-range longitudinal study among 13–21 year-old HPV vaccinated young women from the US found no significant association between risk perceptions after HPV vaccination and high-risk sexual behaviors [18]. Other previous studies from the US and England reported that HPV vaccination was associated with reduction in safer sexual behaviors among youths but didn’t lead to an increased sexual risk-taking behavior [19–21]. There were also previous studies that indicated that HPV vaccination was instead associated to safer sexual behaviors [4, 22–24]. For example, a study conducted among Colombian young women found that HPV vaccination was inversely associated to perceived risk of HPV infection and perceived risk of cervical cancer, was not associated to high-risk sexual behaviors but instead the vaccinated women were more likely to use safer sexual behaviors such as consistent condoms use [23]. Similarly, other studies from the US reported that HPV vaccinated young women were more likely to use condoms than their unvaccinated counterparts [4] and also were no more likely to be treated for an STI than unvaccinated youth [24].

In view of the aforementioned conflicting previous studies on the association between HPV vaccination, CC risk perceptions, high-risk sexual behaviors and STIs, there is need for further research on the topic, more so from developing countries in Africa which carries the heaviest burden of CC. In this study, we compared the HPV-vaccinated young women and their non-vaccinated counterparts in Uganda regarding CC risk perceptions, high-risk sexual behaviors and STIs 5 years after HPV vaccine implementation.

## Methods

### Study design and area

This was a population-based comparative cross sectional study. It was conducted in Ibanda district Uganda as part of a larger HPV16/18 (Cervarix®) vaccine follow up study. Ibanda district was the district in Uganda where the first pilot bivalent HPV 16/18 vaccination project which targeted young women in primary school class 5 (P5) was implemented in 2008.

### Population and sample

The study participants were HPV-vaccinated and non-vaccinated 15–24 year-old young women who were enrolled in secondary schools in Ibanda district. Young women were included in the study if they were sexually active and by 2008 were either in primary school class five (P5) in Ibanda district and were fully vaccinated with 3 doses of the bivalent HPV16/18 vaccine (vaccinated group) or were in primary school class six (P6) or higher in Ibanda district but were not vaccinated with bivalent HPV 16/18 vaccine (non-vaccinated group). Partially vaccinated young women were excluded from the study because they were expected to be very few as the 2008 HPV vaccination coverage in the area was above 95% [25]. The 95% HPV vaccination coverage implied that only 5% of the eligible young girls in the study area were either partially vaccinated (i.e. received 1 or 2 doses of the vaccine) or were not vaccinated at all. At the time of the 5-year follow in 2014, Uganda had not yet adopted the 2-dose regimen for HPV vaccination.

The high HPV vaccination coverage (95%) among the 2008 cohort of girls also made it impractical to establish the required sample size for the non-vaccinated control arm of the study from the same 2008 cohort of girls. Therefore, two different cohorts of girls (i.e. 2006 and 2007 cohorts of the participating schools) were used for the non-vaccinated control group. This however posed the problem of differential age distribution between HPV vaccinated and non-vaccinated control groups.

The sample size was determined by power analysis calculation [26]. Any STI prevalence which was a categorical variable was used as the primary outcome variable for calculating the sample size. The calculation made use of the Normal approximation to the Binomial distribution. The prevalence of any STI in the non-vaccinated young women was assumed to be 10.7% because a previous study conducted in Uganda showed the prevalence of any STI among unvaccinated teenage young women with median age of 20 years was 10.7% [27]. The prevalence of any STI in the vaccinated group that would represent an important improvement from the HPV vaccination was assumed to be 1%. And therefore, the proportions compared in the power analysis calculation were 0.107 and 0.01 and the sample size of 376 was reached at a power of 85% i.e. 85% probability of detecting such a difference, if it really existed, as statistically significant at 5% significance level. This was adjusted to 492 participants (i.e. 241vaccinated and 241 non-vaccinated young women) to cater for potential loss to follow up.

**Fig. 1:**
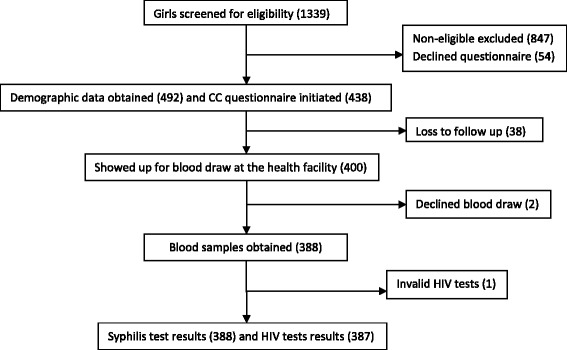
Shows participants flow from enrollment through the study procedures. The study procedures include education about the study, individual counseling and interviewer-administered questionnaire

We performed multi-stage sampling procedure to select the study participants. We first developed a total list of senior secondary schools in Ibanda district where the 2008 cohort of HPV vaccinated young women and their non-vaccinated counterparts were expected to be studying at the time of the study. The total list comprised of 32 secondary schools. Being a school semester period, all the schools in the district were visited one after another by the research team. And young women found in senior secondary school classes three to six (i.e. S3-S6) were approached class by class, informed about the study, consented and screened for eligibility. Demographic data were obtained from all the young women approached. Appointment dates to visit Ruhoko health centre IV, a designated health facility within the district for data/sample collection were agreed with the selected young women and their teachers. On the appointment day, vehicles were sent to the schools to facilitate transportation of the young women to and from the health facility in company of their teachers. Figure 1 shows the flow chart of the participants within the study.

### Data/sample collection

Data/sample collection took place in 2014. At the health facility, data and samples were collected from consecutive young women reaching the facility until the required sample size was reached. The study procedures performed at the health facility included completing the CC questionnaire and collection of blood samples for HIV and syphilis testing.

### Questionnaire

The standard questionnaire was used to collect data about the CC risk perceptions and sexual behaviors (Additional file 1). The questionnaire has two sections namely CC risk perceptions section and sexual behavior section.

The CC risk perceptions section comprised of 16 true or false items namely CC is the same as breast cancer, CC is caused by HPV, HPV is the same as HIV, HPV is transmitted from person to person through sexual intercourse, HPV vaccines when given to virgin young women protects against CC, a virgin young girl should be receive 3 doses of HPV vaccines to be fully protected from CC, women who missed HPV vaccine during their adolescence can still prevent CC by attending regular medical checkup/screening for CC, CC is treatable if detected early, CC is more common among virgin women, women with many sexual partners have a higher chance of developing CC compared to those with one partner, HIV positive women have a higher chance of developing CC compared to HIV negative women, HPV doesn’t infect men, circumcision of men doesn’t reduce the chances of getting infected with HPV, having many sexual partners increases the chances of getting infected with HPV, condom use doesn’t protect against HPV and HPV vaccines also protects against other STDs such as syphilis and gonorrhea. A score of 1 or 0 were awarded for each correct or wrong answer respectively. A sum of scores from the 16-items was obtained for each participant. The range for possible attainable scores was 0–16. A higher score represent higher CC risk perceptions and a lower score represents lower CC risk perceptions. The risk perceptions section of the questionnaire was self-administered under supervision of the study personnel.

Unlike the risk perceptions section questions which were scaled into a composite score, the sexual behavior section questions were used as independent outcome questions, and not scaled into a composite score. The sexual behavior section of the standard questionnaire comprised of 7-items namely age at first sexual intercourse, number of sexual partners in the previous 3 months, number of sexual partners in the previous 12 months, number of sexual partners in the previous 4 years, number of sexual partners in a lifetime, history of STD syndrome (whether she has ever experienced any abnormal vaginal discharge or lower abdominal pain or genital ulcer or groin swelling, warts, vaginal discharge in her life) and level of condom use (whether never, rarely, sometime, often and always). The sexual behavior section of the questionnaire was interviewer-administered to ensure proper phrasing or probing for correct answers and was coupled with health history taking, physical examination and prescription of treatment for those found with health problems.

Both the risk perception and sexual behavior sections of the survey instrument were created by the Study Investigators specifically for the study. Face validity of the instrument was achieved by sending the questionnaire to a sample of 5 experts on HPV vaccination and all of them responded back with the judgement that the scale appears to be a good measure of CC risk perceptions. Armed with the criteria (Additional file 2) for adolescent CC risk perception, the same sample of experts examined the scale for adolescent CC risk perception and all came with the judgement the scale meet the criteria can legitimately be defined as adolescent CC risk perception scale. The reliability index Cronbach’s alpha for the CC risk perception section of the questionnaire which was a scale was 0.67 after accounting for reversely-worded items which was reasonably strong reliability of the scale to measure CC risk perception.

### HIV testing

Fresh venous blood samples drawn from the cubical vein using EDTA vacutainer by two trained/experienced laboratory technicians were used for the HIV tests. HIV-1 testing was performed using first the Determine rapid test (Abbot Diagnostics) [28]. The samples that were not reactive were considered HIV-negative. Otherwise, the Statpak rapid tests (ChemoBio Diagnostics Systems) were used to confirm HIV positivity. In case of disagreement between the 2 tests, a tie-breaker test, the Unigold rapid test (Orgenics), was used. This was the recommended national HIV testing algorithm in Uganda at the time of the study. An independent laboratory technician performed all the HIV testing from Kiwoko health centre IV laboratory in Ibanda district. Young women whose HIV test results turned out to be positive were linked to HIV/AIDS treatment and care program available at the same health facility where all the HIV tests were performed.

### Syphilis testing

Parts of the same venous blood samples drawn for the HIV tests were used for the Syphilis tests. Serum extracted from the venous blood from the laboratory at the study site were kept and transported in liquid nitrogen from the field site to Immunology laboratory at Makerere University College of Health Sciences where they were immediately transferred to minus 80C freezers until testing. The liquid nitrogen tanks ensured cold chain for samples during transportation from fieldwork site to the laboratory. Syphilis tests were performed using a commercially available kit, Human Treponema Pallidum Hemagglutination Assay (TPHA) liquid GmBH, Wiesbaden, German.). The TPHA test is a qualitative microhemagglutination test for the presence of IgG and IgM antibodies to Treponema pallidum, the causative agent for syphilis, in human serum and EDTA plasma. The tests were performed according to the manufacturer’s protocol. Women with positive TPHA test results were referred to clinician for treatment. All the Syphilis tests were performed by yet another independent laboratory technician who wasn’t part of the blood draw team from the Immunology laboratory at Makerere University College of Health Sciences Kampala Uganda.

### Statistical analysis

Data were entered into Statistical Package for Social Sciences (SPSS) version 22.0 for analysis. The HPV-vaccinated and non-vaccinated groups were compared for the demographic data, CC risk perceptions, sexual behaviors and STI prevalence using Pearson’s’ chi-square (x^2^) or Fisher’s exact tests for categorical variables which also generated *p* values, odd ratio (OR) and 95% confidence interval. Continuous variables were compared using independent sample t-test which also provided *p*-values. Additionally, using early age at sexual debut (<16 years vs 16+ years) as the categorical outcome variable, logistic multivariate regression was performed to further examine the association between HPV vaccination and early age at sexual debut after controlling for all other covariates (age, educational level and CC risk perceptions). These covariate included in the logistic multivariate regression were the variables that were significantly associated to early age at sexual debut from the bivariate analysis and were theoretically relevant. Although Uganda national age at sexual debut among females was 15 years [10, 11], in this study, early age at sexual debut cut off was put at <16 because no study participant was aged <15 years.

## Results

The age range of the 438 study participants which comprised of 50.4% HPV-vaccinated young women was 15–24 years and mean age was 18.6 (SD 1.4). All the participants were female students attending secondary school in Ibanda district, the study area in rural southwestern Uganda. All the participants were non-smokers, with neither history of pregnancy, nor child birth, nor abortion nor hormonal contraceptive use. As shown in participants flow schema 1, non-eligibility rate was 63% and the major reason for non-eligibility was sexual inactivity.

As shown in schema 1, of the 492 eligible young women whose demographic data were obtained for the study, 54 declined responding to the CC questionnaire. Of the 438 who initiated responding to the CC questionnaire, 38 didn’t turned up to the health facility for blood draw. Of the 400 who turned up for blood draw, 2 declined blood draw and finally of the 388 blood samples obtained for HIV tests, 1 was invalid for HIV test. The demographic characteristics of the 54 young women who declined the CC questionnaire plus the 38 young women who didn’t show up for blood draw and the 2 young women who declined blood draw were not significantly different from the rest of the study participants who completed the study (statistics not presented here).

### Demographic characteristics of the study participants

Table 1 shows the demographic characteristics of the study participants. With the exception of age, educational level and ethnic tribe, there were no statistically significant differences between HPV-vaccinated and non-vaccinated control groups regarding their demographic characteristics. As shown in Table 1, the non-vaccinated control group was significantly older but also began sexual activity at significantly older age than the HPV-vaccinated group. The age difference between vaccinated and control groups is attributable to the educational level targeted for HPV vaccination in 2008 which was primary school class five irrespective of their age. As shown in Table 1, the HPV-vaccinated young women were significantly younger in age, mostly Ankole by ethnic tribe and mostly from S1-S4 classes compared to the non-vaccinated control young women.

**Table 1:** Demographic characteristics by HPV vaccination status of the participants

		Study groups		
Demographic	Total	HPV-vaccinated	Non-vaccinated	*p*-value
	N	[f(%)]	[f(%)]	
Age in years [mean (sd)]	18.6(sd1.4)	18.1(sd1.2)	19.1(sd1.4)	0.000*
Age group				
• 18+ years	373	373 (44.5)	207(55.5)	0.000*
• <18 years	110	85 (77.3)	25(22.7)	
Address				
• Urban	343	170(67.2)	173(72.4)	0.210
• Rural	149	83(32.8)	66(27.6)	
Ethnic tribe				
• Ankole	358	210(58.7)	148(41.3)	0.000*
• Others	97	31(32.0)	66(68.0)	
Religion				
• Christians	448	238(53.1)	210(46.9)	0.670
• Muslims	5	2(40.0)	3(60.0)	
Education level				
• S1-S4	348	251(72.1)	97(27.9)	0.000*
• S5-S6	134	1(0.7)	133(99.3)	

N is total sample size; f is the frequency; (%) is the percentage; Sd is standard deviation; S1-S4 is senior 1 to senior 4 class; S5-S6 is senior 5 to senior 6 class; * is statistically significant *p* value

### Cervical cancer risk perceptions

As shown in Table 2 (bivariate analysis of the association between cervical cancer risk perceptions and HPV vaccination), the overall cervical cancer risk perceptions among the HPV-vaccinated young women were significantly lower than that among the non-vaccinated young women.

**Table 2:** Bivariate analysis of the Association between Cervical cancer risk perceptions and HPV vaccination

Item	Total	HPV-vaccinated	Non-vaccinated	p
	N	[f(%)]	[f(%)]	
Cervical cancer is the same as breast cancer				
• True	82	45(19.4)	37(18.0)	0.794
• False	356	187(80.6)	69(82.0)	
Cervical cancer is caused by HPV				
• False	48	26(11.3)	22(10.7)	0.969
• True	389	205(88.7)	184(89.3)	
HPV is the same as HIV				
• True	73	38(16.6)	35(17.0)	1.000
• False	362	191(83.4)	171(83.0)	
HPV is transmitted from person to person through sexual intercourse				
• False	42	28(12.1)	14(6.8)	0.085
• True	395	203(87.9)	192(93.2)	
HPV vaccines when given to young girls protect them against cervical cancer				
• False	56	37(16.2)	19(9.2)	0.044
• True	379	192(83.8)	187(90.8)	
A young girl should be given 3 doses of HPV vaccines to be fully protected against cervical cancer				
• False	36	16(7.0)	20(9.7)	0.394
• True	399	213(93.0)	186(90.3)	
Women who missed HPV vaccinations during their adolescence can still prevent cervical cancer by attending regular checkup				
• False	107	59(25.9)	48(23.3)	0.610
• True	327	169(74.1)	158(76.7)	
Cervical cancer is treatable if detected early				
• False	16	10(4.4)	6(2.9)	0.583
• True	419	219(95.6)	200(97.1)	
Cervical cancer is more common among women who have never had sexual intercourse in their lifetime				
• True	104	61(26.3)	43(20.9)	0.223
• False	334	171(73.7)	163(79.1)	
Women with many sexual partners have a higher chance of developing cervical cancer than those with fewer partners				
• False	50	34(14.7)	16(7.8)	0.035
• True	388	198(85.3)	190(92.2)	
More of the HIV positive women develop cervical cancer compared to the HIV negative women				
• False	122	76(32.8)	46(22.3)	0.020
• True	316	156(67.2)	160(77.7)	
HPV doesn’t infect men				
• True	297	159(68.8)	138(67.0)	0.757
• False	140	72(31.2)	68(33.0)	
Circumcision of men doesn’t reduce the chances of getting infected with HPV				
• True	214	114(49.8)	100(48.5)	0.871
• False	221	115(50.2)	106(51.5)	
Having many sexual partners increases the chances of getting infected with HPV				
• False	43	23(10.0)	20(9.7)	1.000
• True	394	208(90.0)	186(90.3)	
Condom use doesn’t protect against HPV				
• False	217	104(45.4)	113(54.9)	0.061
• True	218	125(54.6)		93(45.1)
HPV vaccines also protect against other STDs such as syphilis and Gonorrhea				
• True	177	99(43.2)	78(37.9)	0.298
• False	258	130(56.8)	128(62.1)	
CC risk perception				
• Overall mean score (sd)	12.1(sd 2.1)	11.9(sd 2.3)	12.3(sd 1.8)	0.021*

N is total sample size; CC is cervical cancer; f is the frequency; (%) is the percentage; Sd is standard deviation; * is statistically significant *p* value

In multivariate analysis for the association between low cervical cancer risk perception and set predictors (tribe, religion, address, age at sexual debut and HPV vaccination) as shown in Table 3, HPV vaccination was not significant useful predictor of low cervical cancer risk perception.

**Table 3:** Multivariate Analysis of Predictors for low CC risk perceptions among the young women

	p	OR 95% CI
Tribe		
• Ankole	0.653	0.88 (0.51–1.53)
• Others	ref	
Religion		
• Christians	0.609	1.84 (0.18–19.08)
• Muslims	ref	
Address		
• Urban	0.853	1.05 (0.64–1.71)
• Rural	ref	
Age at sexual debut	0.108	0.94(0.87–1.01)
HPV vaccination status		
• HPV-vaccinated	0.638	1.14(0.66–1.97)
• -vaccinated	ref	

Controlled variables were age and education level. p is *p*-value; OR is Odd ratio; 95% CI is 95% Confidence interval; * is statistically significant *p*-value

### High-risk sexual behaviors

In bivariate analysis (Table 4), there were no significant differences between the HPV-vaccinated and non-vaccinated young women regarding high-risk sexual behaviors with exception of age at sexual debut. The mean age at sexual debut among the HPV-vaccinated young women was significantly lower than that among their non-vaccinated counterparts [15.5 vs. 16.1, p 0.018]. Low at age at sexual debut was used as one of the markers of high-risk sexual behavior among the young girls in this study.

**Table 4:** Bivariate analysis of the Association between sexual behaviors, sexually transmitted infections and HPV vaccination status of the participants

		Study groups		
Item	Total	HPV-vaccinated	Non-vaccinated	*p*
	*N*	[f(%)]	[f(%)]	
Age at sexual debut (mean in years)	15.9(sd2.9)	15.5(sd2.7)	16.1(sd3.1)	0.018*
Age group at sexual debut				
• <16 years	197	68(34.5)	129(65.5)	0.073
• 16+ years	184	47(25.5)	137(74.5)	
Number of sexual partners in previous 3 months				
• 1+ sexual partners	206	113(56.8)	93(50.3)	0.239
• 0	178	86(43.2)	92(49.7)	
Number of sexual partners in previous 1 year				
• 1+ sexual partners	282	150(75.0)	132(71.4)	0.488
• 0	103	50(25.0)	53(28.6)	
Number of sexual partners in previous 4 years				
• 1+ sexual partners	297	153(76.5)	144(77.8)	0.849
• 0	88	47(23.5)	41(22.2)	
Number of sexual partners in a lifetime				
• 2+ sexual partners	65	31(15.5)	34(18.5)	0.521
• 1 partner	319	169(84.5)	150(81.5)	
Condom use				
• Never used	229	121(60.2)	108(58.1)	0.746
• Ever used	158	80(39.8)	78(41.9)	
History of STD syndrome				
• Positive	58	28(13.9)	30(16.1)	0.643
• Negative	329	173(86.1)	156(83.9)	
TPHA (syphilis) test result				
• Positive	4	1(0.5)	3(1.6)	0.351
• Negative	384	202(99.5)	182(98.4)	
HIV test result				
• Positive	7	2(1.0)	5(2.7)	0.264
• Negative	380	201(99.0)		179(97.3)

Sd is standard deviation; TPHA is Treponema pallidum hemagglutination test for syphilis; STI is sexually transmitted infections; STD is Sexually transmitted diseases; N is total sample size; f is frequency; (%) is percentage; p is *p*-value at 5% significance level; * is statistically significant *p*-value

### Factors associated to early age at sexual debut among the participants

As shown in Table 4, at bivariate analysis level, four factors were significantly associated to early age at sexual debut namely having an age less than 16 years, lower educational level [i.e. being senior secondary class one to four (S1-S4)], lower cervical cancer risk perceptions and HPV vaccination.

In multivariate logistic regression analysis level shown in Table 5, it was only age that was a significant predictor of early age at sexual debut [p 0.000, exp.(B) 0.5]. The rest of the factors in the model including HPV vaccination, low education level and low CC risk perceptions were not significant predictors of early age at sexual debut among the participants.

**Table 5:** Multivariate Analysis of Predictors of early age at sexual debut

	p	OR 95% CI
Tribe		
• Ankole	0.664	1.14(0.63–2.07)
• Others	ref	
Religion		
• Christians	0.298	0.33(0.04–2.64)
• Muslims	ref	
Address		
• Urban	0.680	0.90(0.54–1.50)
• Rural	ref	
CC risk perception score	0.010*	0.86(0.77–0.97)
HPV vaccination status		
• HPV-vaccinated	0.869	0.95(0.54–1.68)
• Non-vaccinated	ref	

Variables controlled for were age and education level. p is *p*-value; OR is Odd ratio; 95% CI is 95% Confidence interval; * is statistically significant *p*-value

### Prevalence of syphilis and HIV infections

In bivariate analysis (Table 4), there were no statistically significant differences between the HPV-vaccinated and non-vaccinated young women regarding the prevalence of syphilis and HIV infections. Syphilis and HIV infections were used as some of the bio-markers of high-risk sexual behaviors among the young women in this study.

## Discussion

The HPV-vaccinated young women of our sample were significantly younger in age and were mostly from the lower senior secondary educational level (S1-S4) compared to their non-vaccinated counterparts. This was attributable to the participants sampling strategy used in the study which targeted the 2008 cohort of HPV-vaccinated young women who were expected to be younger and in the lower educational level compared to their non-vaccinated counterparts recruited mostly from the higher senior secondary class five to six (S5-S6).

The ethnic tribal differences between the HPV-vaccinated and non-vaccinated groups observed in the sample for this study could also be attributed to the fact that the 2008 pilot HPV vaccine project in Uganda was implemented in Ibanda district (the study area), a district predominantly occupied by people of Ankole ethnic tribe and hence the HPV-vaccinated young women were more likely to belong to the Ankole ethnic tribe. Notably, the selection of Ibanda district and hence high participation of young women of Ankole ethnic tribe in HPV vaccine rollout program and the study was neither based on HPV and cervical cancer disease burden differences nor cultural differences between ethnic tribes with regard to sexual activity that may impact these findings.

Interestingly, the CC risk perceptions demonstrated by the HPV-vaccinated young women in this study were significantly lower than that for the non-vaccinated control group although it wasn’t significantly associated to high-risk sexual behaviors. This finding was consistent with previous studies from Colombia and US that reported an inverse association between HPV vaccination and perceived risk of CC [18, 23] in bivariate but also not in multivariate analysis. In our sample, the CC risk perceptions differences between the HPV-vaccinated and non-vaccinated groups were attributable to misconceptions such as HPV vaccines doesn’t protect against CC, multiple sexual partners doesn’t increase the risk of CC and HIV infection doesn’t increase the risk of CC which were significantly more prevalent among the HPV-vaccinated group compared to their non-vaccinated counterparts. This finding underscores the importance of educating young women about the risk factors for CC in addition to HPV vaccines as part of vaccine implementation.

With exception of age at sexual debut (data in Table 4), we found no significant differences between HPV-vaccinated young women and their non-vaccinated counterparts regarding high-risk sexual behaviors. Our findings of no association between HPV vaccination and other high-risk sexual behaviors rather than age at sexual debut contradicts with several previous studies that reported that HPV-vaccinated young women were more likely to experience sexual intercourse [14] or were more likely to have positive attitudes toward maintaining safe sexual health [22] or were more likely to use safer sexual behaviors such as consistent condoms use [23] or were more likely to use condoms [4] or were no more likely to be treated for an STI than the unvaccinated youth [24]. On the other hand, our finding of no association between HPV vaccination and other high-risk sexual behaviors concurs with some previous studies including in US and Canada that similarly indicated no association between HPV vaccination and high-risk sexual behaviors among young women [12–18]. In view of the above, our finding in agreement with more recent previous studies doesn’t support the possibility of sexual disinhibition among young women following HPV vaccination.

Our finding regarding the association of HPV vaccination with early age sexual debut in bivariate analysis although not significant in multivariate analysis contracts with a number of previous studies from other parts of the world that reported no association between HPV vaccination and early age at sexual debut in both bivariate and multivariate analyses [&, 11, 12, 14, 22]. In our sample, the association between HPV vaccination and early age at sexual debut was probably confounded by other covariates among the participants such that in multivariate model containing other covariates, HPV vaccination was found to be not a significant predictor of early age at sexual debut but age. This could imply that young women of younger generation (vaccinated group) starts sexual activity at an earlier age than young women of older generation (non-vaccinated group), emphasizing the importance of initiating HPV vaccination of young women at a younger age than 15 years before they start sexual activity.

The HPV-vaccinated young women in our sample didn’t differ significantly from their non-vaccinated counterparts regarding the history of STD syndrome and the prevalence of both syphilis and HIV infections. These findings from our study were consistent with previous studies from the US and Sweden that both reported no significant differences between HPV-vaccinated and their non-vaccinated counterparts regarding STI prevalence rates [12, 14]. Since STIs are often used as objective biomarker of safer sexual behaviors, our findings in line with previous studies regarding no association between HPV vaccination and STIs further suggest that HPV vaccination doesn’t have detrimental effect on sexual health of young women.

The strengths of our study include being one of the first HPV vaccine follow up studies conducted from developing country in Africa with high burden of HPV-related cancers to empirically examine the association between HPV vaccination, CC risk perceptions and high-risk sexual behaviors using a population-based comparative cross sectional survey design. We assessed for sexual behaviors both subjectively using a questionnaire and objectively by performing laboratory tests for syphilis and HIV infections which were the respectable biomarkers of high-risk sexual behaviors used in previous related studies among young women [12, 14]. And we made use of non-vaccinated control group and multivariate logistic regression to control for extraneous variables.

A weakness of our study was that some of the data were self-reported and hence subject to reporting bias. We minimized on reporting bias by using a standard questionnaire and ensuring that the sexual behavior section of the questionnaire was interviewer-administered which provided opportunities for clarification on questions and probing. Another potential weakness was the fact that we studied a largely rural in-school young women population shortly after their sexual debut and hence our results may not be widely applicable to a more diverse population.

Further research on this topic in developing country particularly Africa should be a long term HPV vaccine follow up of the young women for cervical cancer secondary prevention outcomes such as attitudes and uptake of cervical cancer screening.

## Conclusions

We found that CC risk perceptions and age at sexual debut were both significantly lower among the HPV-vaccinated young women compared to their non-vaccinated counterparts in Uganda 5 years after vaccine implementation. However, HPV vaccination was neither associated to lower cervical cancer risk perceptions nor early age at sexual debut in multivariate analysis. Other high-risk sexual behaviors were rampant among both groups although the differences were not statistically significant. Our findings don’t support the notion that HPV vaccination is associated with high-risk sexual behaviors. Importantly, we found that young women in study area in rural Uganda were sexually active at a younger age and are engaged in high-risk sexual behaviors, affirming the importance of targeting young women of younger age for HPV vaccination.

## Additional file

Additional file 1: Questionnaire. (DOCX 52 kb)
Additional file 2: STROBE Statement—checklist of items that should be included in reports of observational studies. (DOC 109 kb)

## Abbreviations

CC: Cervical cancer
EDTA: Ethylene diamine tetra-acetic acid
HIV: Human Immunodeficiency Virus
HPV: Human Papillomavirus
IgG: Immunoglobulin G
IgM: Immunoglobulin M
P5: Primary school class 5
P6: Primary school class 6
S3: Senior secondary school class 3
S4: Senior secondary school class 4
S5: Senior secondary school class 5
S6: Senior secondary school class 6
SD: Standard deviation
STDs: Sexually transmitted disease(s)
STIs: Sexually Transmitted Infection(s)
TPHA: Treponema Pallidum Hemagglutination
