# Supplementary material for: Cervical cancer risk perceptions, sexual risk behaviors and sexually transmitted infections among Bivalent Human Papillomavirus vaccinated and non-vaccinated young women in Uganda - 5 year follow up study
Source: BMC Womens Health. 2017 Jun 2;17:40. doi: 10.1186/s12905-017-0394-y (PMC5457617; doi:10.1186/s12905-017-0394-y)
Supplement: Additional file 1: — Questionnaire. (DOCX 52 kb) [file 12905_2017_394_MOESM1_ESM.docx]

**QUESTIONNAIRE**

Study ID: ________________________ Study arm: ____________________

**SECTION A: SOCIO-DEMOGRAPHIC FORM**

Study ID _______________________

1. Age: ___(by verbal report) ___(by HPV card) __(by HPV register)
2. Date of birth: ___(verbal report) ____(HPV card) ___(HPV register)
3. Current residential adress: _____________________________
4. Religion: ____________________
5. Tribe: _________________________
6. Education level or current class in school: ___________________
7. Occupation (if out-of-school): _____________________________
8. Marital status (if out-of-school): ___________
9. Class in 2008 or 2009 ______ (verbal report) ______(HPV card) ______(by HPV register)
10. District in 2008 or 2009 _____ (by girls verbal report) ____(by HPV card) ___(by HPV register)
11. # doses of HPV vaccine received: ______(by girls verbal report) ____(by HPV card) ___(by HPV register)
12. Dates for: HPV1______HPV2_________HPV3_____ (verbal report)
13. Dates for: HPV1______HPV2_________HPV3_____ (HPV card)
14. Dates for: HPV1___HPV2_____HPV3____ (HPV register)
15. Name of HPV vaccination post: _________ (verbal report) ____(by HPV card) ___(by HPV register)
16. Final Decision on study arm: ___________________
17. Batch #: HPV1_____HPV2____HPV3____ (by vaccine control books)

**SECTION B: CERVICAL CANCER RISK PERCEPTIONS**

| **PERCEPTION ITEM** | True (T) OR  False (F) | Score  0 OR 1 |
| --- | --- | --- |
| Cervical cancer is the same as breast cancer |  |  |
| Cervical cancer is caused by HPV |  |  |
| HPV is the same as HIV |  |  |
| HPV is transmitted from person to person through sexual intercourse |  |  |
| HPV vaccines when given to young girls protect them against cervical cancer |  |  |
| A young girl should be given 3 doses of HPV vaccines to be fully protected against cervical cancer |  |  |
| Women who missed HPV vaccinations during their adolescence can still prevent cervical cancer by attending regular check-up |  |  |
| Cervical cancer is treatable if detected early |  |  |
| Cervical cancer is more common among women who have never had sexual intercourse in their lifetime |  |  |
| Women with many sexual partners have a higher chance of developing cervical cancer than those with fewer partners |  |  |
| More of the HIV positive women develop cervical cancer compared to the HIV negative women |  |  |
| HPV doesn’t infect men |  |  |
| Circumcision of men doesn’t reduce the chances of getting infected with HPV |  |  |
| Having many sexual partners increases the chances of getting infected with HPV |  |  |
| Condom use doesn’t protect against HPV |  |  |
| HPV vaccines also protect against other STDs such as syphilis and Gonorrhea |  |  |
| Total CC risk perception score |  |  |
|  |  |  |

**SECTION C: SEXUAL BEHAVIOR QUESTIONNAIRE**

Study ID _______________________

1. Age at diagnosis or treatment for STIs/STDs ________ years
2. Signs and symptoms of STIs/STDs _________________________
3. HIV status____________
4. Age at HIV diagnosis______ years
5. Age at initiation of ART __________ years
6. Age at sexual debut: _______________years
7. Number of sexual intercourse in the last 3 months _____________
8. Number of sexual partners in the last 3 months______________
9. Number of sexual intercourse in the last 1 year________________
10. Number of sexual partners in the last 1 year __________________
11. Number of sexual intercourse in the past 4 years ? _________
12. Number of sexual partners in the past 4 years? ____________
13. Number of life time sexual partners? ___________________

**The end, thank you for your participation.**
